# High optode-density wearable diffuse optical probe for monitoring paced breathing hemodynamics in breast tissue

**DOI:** 10.1117/1.JBO.26.6.062708

**Published:** 2021-06-02

**Authors:** Samuel S. Spink, Fei Teng, Vivian Pera, Hannah M. Peterson, Tim Cormier, Alexis Sauer-Budge, David Chargin, Sam Brookfield, Adam T. Eggebrecht, Naomi Ko, Darren Roblyer

**Affiliations:** a Boston University, Department of Biomedical Engineering, Boston, Massachusetts, United States; b Boston University, Department of Electrical and Computer Engineering, Boston, Massachusetts, United States; c Boston University, Fraunhofer Center for Manufacturing Innovation, Boston, Massachusetts, United States; d Washington University, Department of Radiology, St. Louis, Missouri, United States; e Boston Medical Center, Section of Hematology and Oncology, Women’s Health Unit, Boston, Massachusetts, United States

**Keywords:** diffuse optics, diffuse optical imaging, wearable, breast cancer, paced breathing, hemodynamics

## Abstract

**Significance:** Diffuse optical imaging (DOI) provides *in vivo* quantification of tissue chromophores such as oxy- and deoxyhemoglobin (${\mathrm{HbO}}_{2}$ and HHb, respectively). These parameters have been shown to be useful for predicting neoadjuvant treatment response in breast cancer patients. However, most DOI devices designed for the breast are nonportable, making frequent longitudinal monitoring during treatment a challenge. Furthermore, hemodynamics related to the respiratory cycle are currently unexplored in the breast and may have prognostic value.

**Aim:** To design, fabricate, and validate a high optode-density wearable continuous wave diffuse optical probe for the monitoring of breathing hemodynamics in breast tissue.

**Approach:** The probe has a rigid-flex design with 16 dual-wavelength sources and 16 detectors. Performance was characterized on tissue-simulating phantoms, and validation was performed through flow phantom and cuff occlusion measurements. The breasts of N=4 healthy volunteers were measured while performing a breathing protocol.

**Results:** The probe has 512 unique source–detector (S-D) pairs that span S-D separations of 10 to 54 mm. It exhibited good performance characteristics: $\mu_{a}$ drift of 0.34%/h, $\mu_{a}$ precision of 0.063%, and mean SNR≥24  dB up to 41 mm S-D separation. Absorption contrast was detected in flow phantoms at depths exceeding 28 mm. A cuff occlusion measurement confirmed the ability of the probe to track expected hemodynamics *in vivo*. Breast measurements on healthy volunteers during paced breathing revealed median signal-to-motion artifact ratios ranging from 8.1 to 8.7 dB. Median $\Delta {\mathrm{HbO}}_{2}$ and ΔHHb amplitudes ranged from 0.39 to 0.67  μM and 0.08 to 0.12  μM, respectively. Median oxygen saturations at the respiratory rate ranged from 82% to 87%.

**Conclusions:** A wearable diffuse optical probe has been designed and fabricated for the measurement of breast tissue hemodynamics. This device is capable of quantifying breathing-related hemodynamics in healthy breast tissue.

## 1 Introduction

An estimated 84,000 new cases of locally advanced breast cancer (LABC) will be diagnosed in 2021 (this estimate refers to the number of projected “regional” breast cancer cases, which has significant overlap in definition with LABC).^1^ The ability to predict neoadjuvant chemotherapy (NAC) treatment response for these patients is considered an important unmet clinical need, especially as NAC becomes increasingly common.^2^ Accurate treatment response monitoring could lead to adaptive therapies, in which treatment strategy is dynamically altered as a function of treatment response to improve outcomes and reduce the burden of toxic side effects on patient. As noted by Laura Esserman in a recent *Clinical Cancer Research Translation* article, a benefit of the neoadjuvant setting is that “we can rapidly evaluate new and promising therapies, and escalate if treatment is suboptimal, avoiding additional toxic therapy if responses are excellent.”^3^ Of course, this necessitates technologies capable of tracking response longitudinally during treatment.

Diffuse optical imaging (DOI), also commonly referred to as near-infrared spectroscopy, or NIRS, is a label-free, noninvasive, and relatively inexpensive functional imaging modality that has been explored as a method of predicting NAC treatment response in the clinic. DOI technologies are used to quantify concentrations of oxy- and deoxyhemoglobin (${\mathrm{HbO}}_{2}$ and HHb), $H_{2}O$, and lipid content in tissue. Measurement penetration depth can extend up to several centimeters, allowing for functional imaging of subcutaneous tumors.^4^ The performance trade-off comes primarily in the form of spatial resolution, which, depending on the number of light sources and detectors and their geometry, can range from ∼0.5 to 10 mm.^5^^,^^6^ While time-domain (TD) and frequency-domain (FD) DOI devices enable absolute quantification of absorption and scattering coefficients and chromophore concentrations, continuous wave (CW) systems can quantify only relative changes in absorption and chromophore concentrations but are simpler and less expensive. Many prior studies have utilized FD-DOI or TD-DOI to obtain absolute concentrations of ${\mathrm{HbO}}_{2}$, HHb, $H_{2}O$, and lipid content at baseline and time points leading up to the midpoint of NAC.^7^^–^^13^ These studies have generally found that decreases in HHb and $H_{2}O$ and an increase in lipid at various time points between 1 week and midpoint of chemotherapy (compared to baseline) are well correlated with a pathologic complete response (pCR). One of these studies even showed statistically significant optical contrast in ${\mathrm{HbO}}_{2}$ between responders and nonresponders as early as 1 day into chemotherapy.^9^

There are a number of drawbacks to existing DOI technologies for monitoring breast cancer treatment response. Devices typically require an operator to scan a handheld probe in a point-by-point manner over breast tissue,^7^^–^^10^^,^^13^ or the patient to insert herself into a fixed, rigid optical fiber array in a prone position or while bending over.^11^^,^^12^^,^^14^^–^^16^ These configurations may be awkward for the patient due to the required body position and/or the contact with an operator. The handheld probe is portable to an extent but requires laborious point-by-point scanning. Tomographic fixed arrays of sources and detectors allow for better temporal resolution with high spatial information, but portability and flexibility are limited, they require more space for usage and storage, and patients may be less willing to attend multiple imaging sessions. Wearable and portable DOI and NIRS technologies solve several of these issues, but most current technologies suffer from either low optode-density or poor mechanical fit to breast tissue, limiting their application in the monitoring of breast tumor hemodynamics.^17^^–^^20^ We have previously developed a wearable probe that offers improved breast tissue contact by incorporating a rigid-flex-printed circuit board (PCB),^21^ but this device was limited as it had only 12 sources and 1 detector that all share the same source–detector (S-D) separation.

In this work, we developed a high optode-density wearable CW probe with 32 LED sources and 16 detectors. It is both mechanically flexible and able to provide high spatial resolution and is suited to the dimensions of most locally advanced breast tumors.^22^ We leveraged this new probe to explore paced breathing hemodynamics in breast tissue for the first time. It has been previously shown that blood volume oscillations at the respiratory rate during paced breathing can be detected in the brain and in periphery.^23^^,^^24^ Such oscillations have also been used to extract venous oxygen saturation in pigs, the thighs of human subjects, and in the brains of neonates.^25^^,^^26^ We hypothesize that metrics derived from these oscillations may prove to be sources of contrast between cancerous and healthy tissue in the breast. In addition, compared to breath holds, which have recently been explored in the context of NAC monitoring,^15^^,^^16^ paced breathing offers a potentially more patient-friendly protocol, as it is easier to perform.

In the following sections, we first describe the design and fabrication of the new high optode-density wearable probe. We then describe system characterization results, followed by both *in vitro* and *in vivo* validation through measurements on spatially complex optical phantoms and the forearm of a healthy volunteer during a cuff occlusion. We then quantify breathing motion artifact, and lastly, quantify paced breathing hemodynamics in breast tissue through measurements on a group of four healthy volunteers.

## 2 Instrument Design

Factors considered in the design of the wearable probe included the tissue depth and sizes of locally advanced breast tumors, the near-infrared (NIR) absorption and scattering properties of human breast tissue, and the anticipated hemodynamic changes expected to occur. Design choices, methods, and results are described below.

### 2.1 Wavelength Selection

The probe was designed to extract ${\mathrm{HbO}}_{2}$ and HHb concentration changes. The choice of imaging wavelengths was determined in part by a condition number analysis previously shown to be useful for optimizing wavelength.^27^ The condition number κ(A) is defined as

$$\kappa (A)=\frac{\sigma_{\max}(A)}{\sigma_{\min}(A)},$$ (1)

where $A=(\begin{array}{cc} \varepsilon_{\lambda_{1}}^{{\mathrm{HbO}}_{2}} & \varepsilon_{\lambda_{1}}^{\mathrm{HHb}}\\ \varepsilon_{\lambda_{2}}^{\mathrm{Hb}O_{2}} & \varepsilon_{\lambda_{2}}^{\mathrm{HHb}} \end{array})$, the elements of A are the optical extinction coefficients for ${\mathrm{HbO}}_{2}$ and HHb at the chosen wavelength pair, and $\sigma_{\max}(A)$ and $\sigma_{\min}(A)$ are the maximum and minimum singular values of A. Extinction coefficients were taken from Ref. 28. Optimized wavelength pairs correspond to a condition number closer to one. Dual-wavelength pairs in the NIR wavelength band from 600 to 900 nm were tested using condition number analysis. 750- and 850-nm LEDs were chosen for the wearable probe based on their relatively small condition number (3.1) as well as the commercial availability of LEDs at these wavelengths.

### 2.2 Optode Topology

The imaging probe was designed to measure the most prevalent tumor size ranges based on current epidemiological data, with a target range of 2 to 4 cm in diameter, which represent the 34 and 74 percentiles of locally advanced breast tumors sizes.^22^ An overall probe diameter of 60 mm was chosen for all designs to accommodate a large range of tumor sizes.

**Fig. 1:**
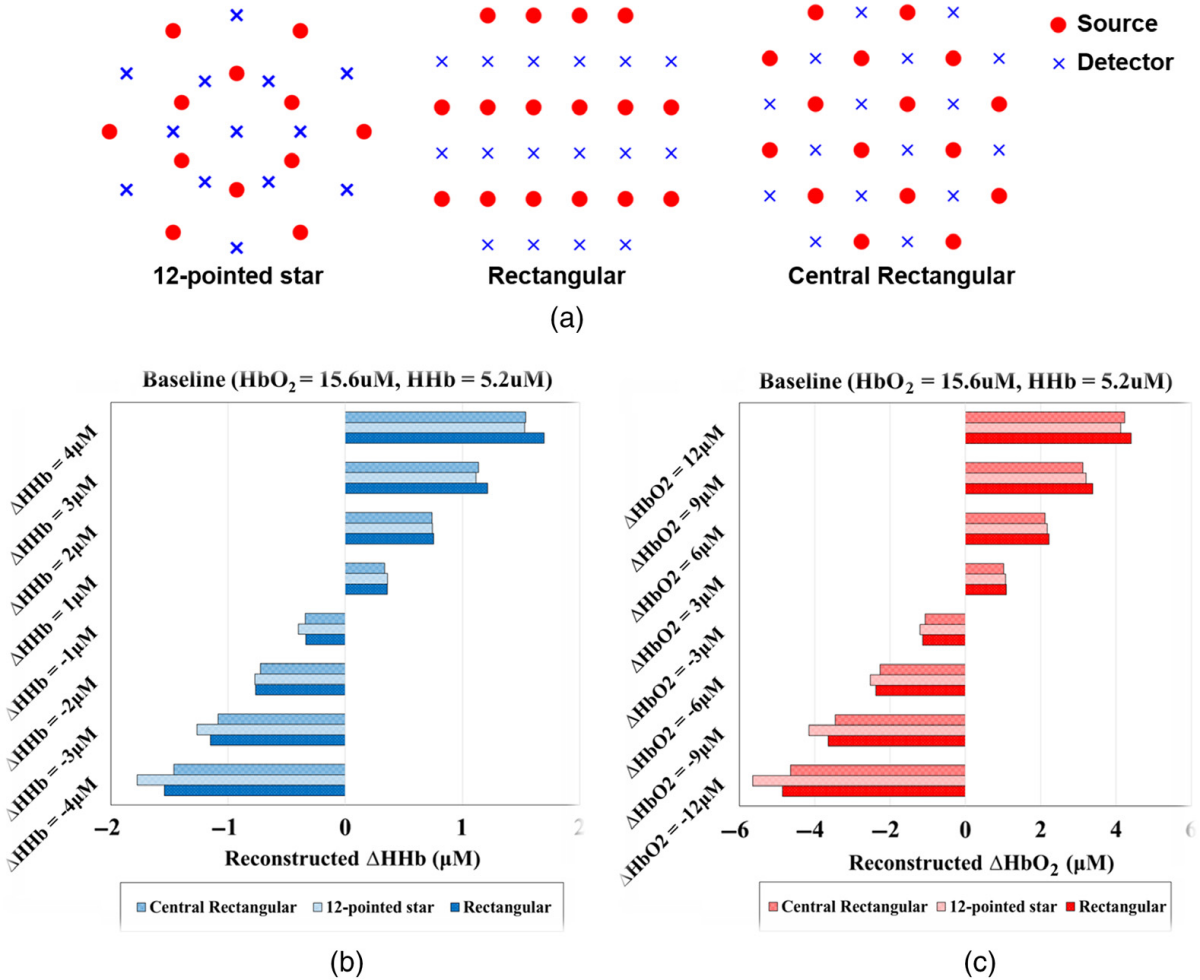
(a) Simulated optode topologies. Tomographic reconstruction results for (b) ΔHHb and (c) $\Delta {\mathrm{HbO}}_{2}$ from various simulated chromophore perturbations in an embedded inclusion (tumor). Simulation ground truth is indicated on the y-axis and reconstructed values are indicated on the x-axis.

Three optode topographies were considered and compared using finite element method (FEM) simulations of a breast tumor hemodynamic challenge. The topographies are shown in Fig. 1(a) and included a “12 pointed star” pattern, a “rectangular” pattern, and a “central rectangular” pattern. The “12-pointed star” pattern was an extension of our prior probe design, which utilized a central hub with arms radiated outward.^21^ Both the rectangular and central rectangular topographies have been utilized in prior tomographic NIRS systems for neuroimaging applications.^29^^–^^32^

Each probe optode topology was tested using Nirfast, a Matlab software suite that utilizes FEM for numerically solving the photon diffusion equation.^33^ A tumor-like inclusion was modeled on a homogeneous background and incremental increases and decreases in the inclusion absorption were simulated. The background ${\mathrm{HbO}}_{2}$ and HHb concentrations were set to 15.6 and 5.2  μM, respectively, and were based on previously reported healthy breast tissue properties.^34^ The inclusion was a 35-mm diameter sphere at a depth of 5 mm. Figure S1 in the Supplemental Material depicts this simulation geometry. ${\mathrm{HbO}}_{2}$ was incremented or decremented by 3, 6, 9, and 12  μM and HHb was incremented or decremented by 1, 2, 3, or 4  μM. The magnitude of these changes roughly corresponds to the documented changes of chromophore concentrations for locally advanced breast tumors on the day one after their initial NAC infusion.^9^ The reconstructed $\Delta {\mathrm{HbO}}_{2}$ and ΔHHb concentrations were compared with the ground truth for all simulations. Crosstalk between reconstructed $\Delta {\mathrm{HbO}}_{2}$ and ΔHHb was also evaluated. Scattering was assumed to be homogeneous and set to a wavelength-insensitive constant of $1\;{\mathrm{mm}}^{-1}$ for all simulations, which is a common reduced scattering coefficient for breast tissue.^35^

Reconstructed chromophore changes were calculated within the three-dimensional (3D) inclusion volume for FEM simulations conducted with each optode geometry. Results are shown in Figs. 1(b) and 1(c). Results were similar for each geometry with 35% to 40% of target changes recovered, meaning all geometries produced an underestimation of reconstructed $\Delta {\mathrm{HbO}}_{2}$ and ΔHHb. A 6% to 12% crosstalk was observed between ΔHHb and $\Delta {\mathrm{HbO}}_{2}$. The “central rectangular” pattern was chosen for the probe design because of the equivalent simulation results and because of its relative ease of fabrication. This design has seven S-D separation orders, ranging from 10 to 54 mm. There is a total of 512 unique S-D pairs.

### 2.3 Printed Circuit Board Design

**Fig. 2:**
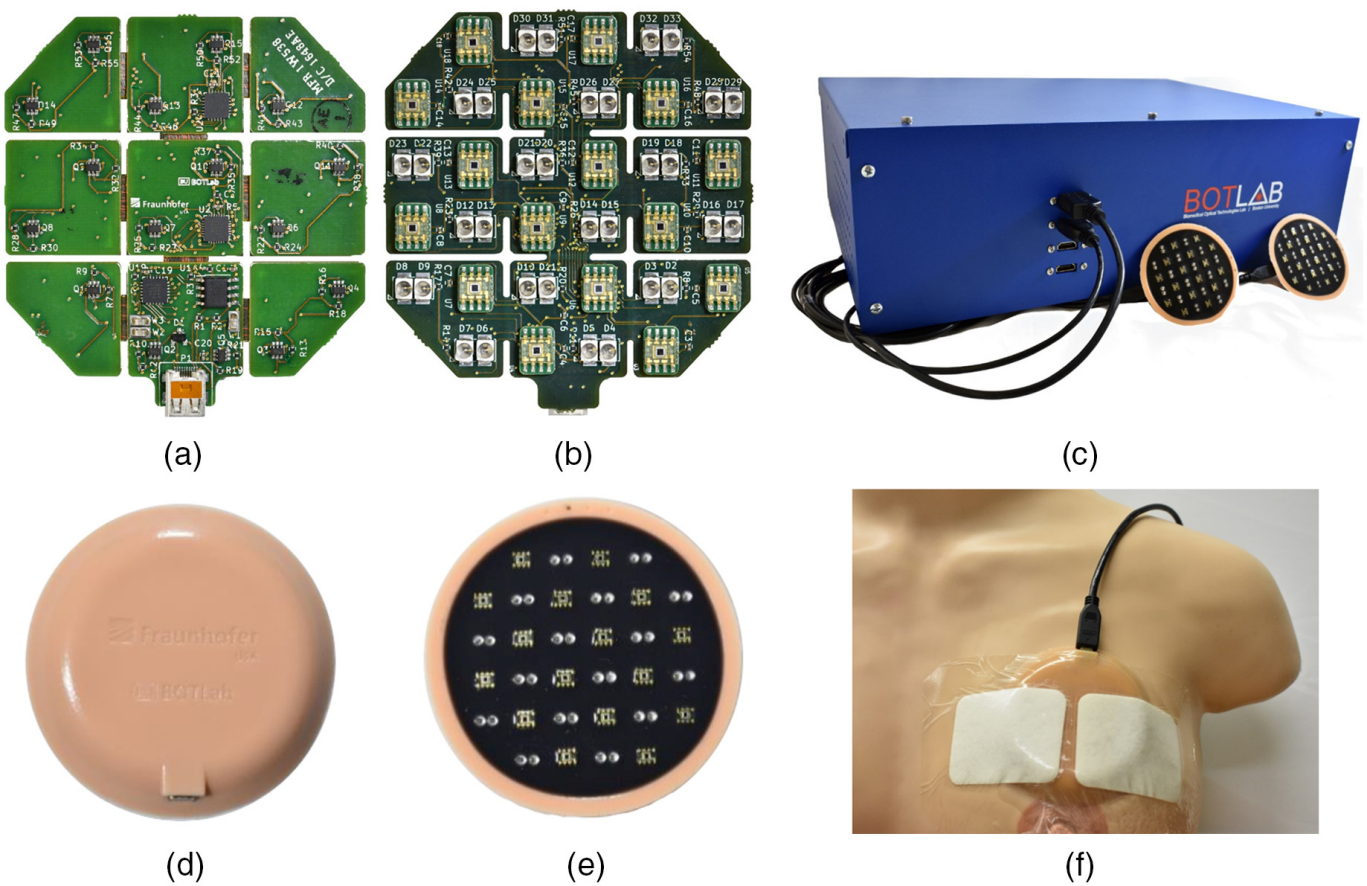
(a) Bottom and (b) top copper layers of rigid-flex PCB, as well as (c) back and (d) tissue-facing sides of encased probe. The dimensions of the bare PCB are ∼6  cm×6  cm, and the encased probe is 8 cm in diameter and 1 cm thick. (e) The peripheral control box with two probes connected via HDMI cable. The dimensions of the box are 42.9  cm×32.5  cm×14.5  cm. (f) The wearable probe attached to the curved surface of human bust model.

The probe is shown in Fig. 2. It was fabricated using a four-layer rigid-flex PCB design. Nine rigid PCB “islands” are connected through 0.9-mm flexible connections that allow flexion between rigid neighbors. The bottom copper layer [shown in Fig. 2(a)] is populated with several $I^{2}C$ compatible integrated circuits including an LED driver (MAX6964, Maxim Integrated), a bus buffer (PCA9600, NXP Semiconductors), and two analog multiplexers (MAX14661, Maxim Integrated). In addition, MOSFET arrays (SSM6N43FU, LF, Toshiba) are used to sequentially switch each LED. The middle layers of the PCB sit at the interface between the rigid and flexible substrates and facilitate trace routing.

The top copper layer [shown in Fig. 2(b)] is populated with 32 LEDs (16 SMT750-23 and 16 SMT850-23, Roithner Lasertechnik) and 16 optical detectors (TSL250RD, ams). The LEDs are surface mount PLCC-2 packages with 750- and 850-nm center wavelengths. The detector is an 8-SOIC silicon photodetector module with a built-in transimpedance amplifier, and the maximum responsivity of this sensing module is $76.8\;\mathrm{mV}/(\mu W/{\mathrm{cm}}^{2})$ at 770 nm.

The wearable probe communicates with a peripheral control box through an HDMI cable. A flexible ribbon micro-HDMI to micro-HDMI adapter cable connects directly to the probe, and then a more rigid micro-HDMI to HDMI cable connects the ribbon cable to the control box. The peripheral control box [shown in Fig. 2(c)], powered externally by connection to a wall outlet, contains a DAQ board (USB-6361 OEM, National Instruments) with a 16-bit analog-to-digital converter, a current controller (FL591), and an Aardvark $I^{2}C$ adapter (TP240141, Total Phase). A custom Labview program on a PC sends commands via USB to both the DAQ board and Aardvark adapter. The Aardvark adapter communicates with the wearable probe via HDMI to control the sequential scanning of optodes, and analog signal from the probe is relayed back to the DAQ board, which digitizes the signal and sends it to the PC for data saving and display.

### 2.4 Biocompatible Probe Housing

The probe is designed to conform to the curved surface of breast tissue and is potted and cured in a medical grade silicone housing. Figures 2(d) and 2(e) show the top and bottom sides of the probe encased in housing. It is made with skin-safe silicone (Ecoflex 00-30, Smooth-On Inc.) and pigments (Silc Pig, Smooth-On Inc.). The silicone and curing agents are mixed with the color dye, vacuumed, transferred to a 3D-printed mold, further vacuumed, and then cured. A black silicone layer is used on the bottom surface of the probe to reduce light crosstalk from emitters to detectors. The optical emitters and detectors protrude from the silicone surface by ∼0.3  mm and indent the soft tissue of the breast. The total dimension is 8 cm wide and 1 cm thick, and its overall weight is 41.8 g. Surgical IV tape firmly secures the silicone probe over the curved surface of bust model as shown in Fig. 2(f).

## 3 System Characterization and Validation

### 3.1 Performance Testing

**Fig. 3:**
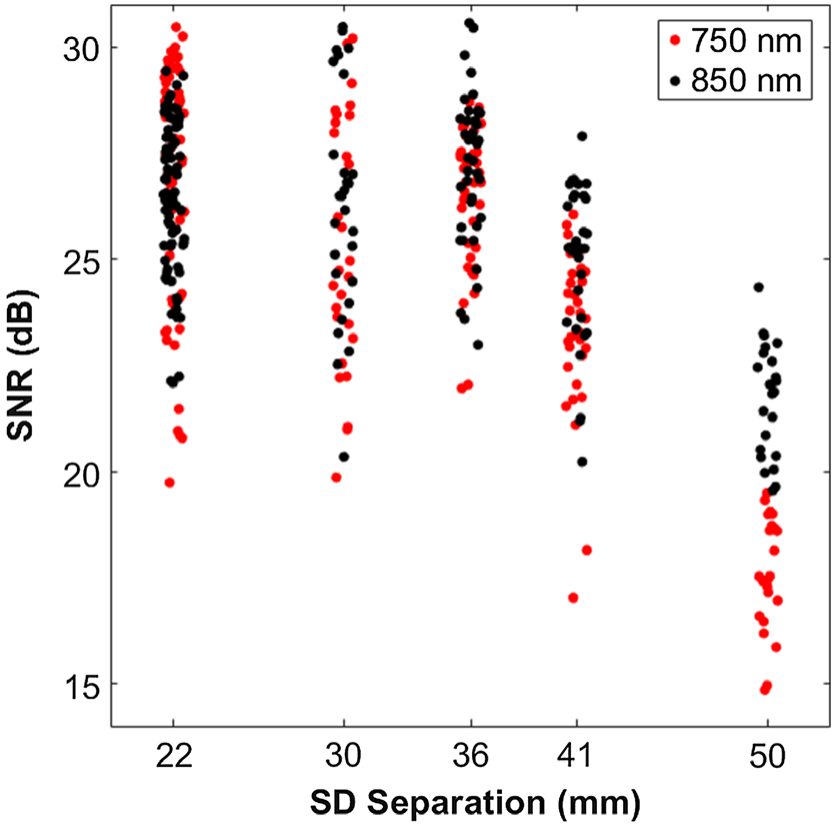
Signal to noise for probe SD separations. Horizontal displacement (jitter) within an S-D separation was added for ease of visualization and does not reflect true S-D separation variation.

The SNR of each S-D pair was evaluated using a custom silicone tissue-simulating phantom with optical properties that closely match reported healthy breast tissue values.^34^^,^^35^ These optical properties were confirmed using a benchtop frequency-domain diffuse optical spectroscopy (FD-DOS) system^36^ and were measured to be: $\mu_{a}=0.006$ (750 nm), $\mu_{a}=0.005{\;\mathrm{mm}}^{-1}$ (850 nm), and $\mu_{s}^{\prime }=0.743{\;\mathrm{mm}}^{-1}$ (750 nm), $\mu_{s}^{\prime }=0.635{\;\mathrm{mm}}^{-1}$ (850 nm). Uncertainties for these (and subsequent) FD-DOS measurements are $∼0.001\;{\mathrm{mm}}^{-1}$, based on our prior work.^37^ SNR was calculated according to

$$\text{SNR}=10\cdot \log_{10}(\frac{\text{mean}(V_{p})-\text{mean}(V_{d})}{\mathrm{std}(V_{p})}),$$ (2)

where $V_{p}$ and $V_{d}$ are the DC voltages collected for phantom and dark measurements, respectively. Phantom measurements were taken over the course of ∼1  h sampled at 0.2 Hz, whereas five dark measurements were taken sequentially at 0.2 Hz, prior to the phantom measurements. Dark measurements were taken with the LEDs off, and all measurements were taken under ambient fluorescent lighting. Figure 3 shows the results of this characterization, with mean SNR values of 27, 26, 27, 24, and 20 dB at the S-D separations of 22, 30, 36, 41, and 50 mm. SNR was at least 15 dB for all S-D pairs. At each separation order, SNR varied by as much as 11 dB, likely due to differences in optical coupling between the optical transducers and phantom.

We note that SNR and other performance metrics were determined for the second- to sixth-order of optode separation, or roughly 22 to 50 mm separations. This included 392 S-D pairs (as opposed to all 512). These S-D pairs were typically used for physiological measurements, as first- and seventh-order optodes often experience optical detector saturation and low signal, respectively, on breast tissue. We also note that within each separation order the exact separation can vary up to ∼3  mm due to the placement of the LEDs on the PCB. Optodes of the same separation order are grouped for ease of visualization, labeled with the mean separation. For example, the second-order, which refers to optodes whose separations range from ∼21 to ∼24  mm, will be labeled “22 mm.”

Other system performance characteristics are presented in Table 1. Dynamic range, drift, and precision metrics refer to the mean of the given metric across all 392 S-D pairs and were computed based on data collected during the same measurement described for the SNR calculation. Dynamic range was computed as the maximum measurable voltage of the photodiode (3.7 V) divided by the standard deviation of five sequential dark measurements.

**Table 1:** System characteristics of high-optode density wearable probe. Dynamic range, drift, and precision refer to mean values across all 392 S-D pairs.

Dimensions and optodes		Performance characteristics	
Diameter	80 (mm)	Dynamic range	68 (dB)
Thickness	10 (mm)	Acquisition rate	0.4 (Hz)
Wavelength	750/850 (nm)	Drift ($\mu_{a}$)	0.34 (%/h)
Total # S-D pairs	512	Precision ($\mu_{a}$)	0.063 (%)
Orders of optode separation	7	Thermal effect^a^	0.29 (%/°C)

a Cited from our prior work.^21^

For system drift and precision calculations, changes in voltages were first converted to $\Delta \mu_{a}$ values using the modified Beer–Lambert law (MBLL),^38^ with the differential pathlength factor (DPF) computed based on the initial optical properties measured with the benchtop frequency-domain system and the S-D separation.^39^ Drift was calculated by computing the slope between the two endpoints of the 1-h phantom measurement, with the starting point being the mean of the first 10 samples (∼first 50 s) and the end point being the mean of the last 10 samples (∼last 50 s). Slope values, in units of ${\mathrm{mm}}^{-1}/h$, were then normalized by the initial $\mu_{a}$ values at each wavelength to obtain percent change in $\mu_{a}$ (%/h).

System precision was computed by first band-pass filtering the temporal $\Delta \mu_{a}$ and then calculating the standard deviation of the band-pass filtered signal. Precision was then reported as a coefficient of variation (%) by normalizing the standard deviation by the initial $\mu_{a}$ values each wavelength. The band-pass filter used was a fifth-order Butterworth filter with cut-off frequencies of 0.045 and 0.055 Hz. This filter was selected to determine precision at 0.05 Hz, which is the breathing frequency of particular interest in subsequent physiological breast measurements.

The acquisition speed of the probe was determined by recording the time needed to sequentially scan the 392 S-D pairs of one probe. Normal operation of this system, however, involves the sequential scanning of two probes, so most measurements are acquired at a rate of 0.2 Hz, as opposed to 0.4 Hz. It should be noted that the forward current to LEDs can be dynamically altered to accommodate different S-D separations, and that the degree of forward current switching can alter acquisition speed. The cuff occlusion experiment described in Sec. 3.3 required no forward current switching, resulting in a higher acquisition rate of 0.33 Hz. Thermal characteristics shown in Table 1 are cited from our prior work.^21^

### 3.2 *In Vitro* Validation with Flow Phantoms

Flow phantom experiments were performed to validate the ability of the wearable probe to quantify absorption at a range of depths and to localize absorption contrast spatially. A solid breast tissue mimicking phantom was fabricated (same optical properties as in Sec. 3.1) that had three 10-mm-diameter hollow channels at three depths. The depths were 7, 19, and 28 mm, with these depths referring to the distance between the surface of the solid phantom and the upper edge of the channel. The centers of the channels were laterally displaced by ∼20  mm. Flow phantom experiments were performed by initially prefilling the channels with a solution of 1% Intralipid in water, and then injecting a second solution containing 1% Intralipid, water, and nigrosin dye at a concentration of 0.05  mg/ml into one of the three channels. Figure S2 in the Supplemental Material depicts this flow phantom layout. The probe was adhered to the surface of the solid phantom with IV tape, centered above the channel through which the second solution was flowing. Baseline measurements were recorded for ∼0.5  min at 0.2 Hz, after which a syringe pump controlled the dispensing of the nigrosin solution into one of the channels at a continuous rate while the probe continued to record measurements for ∼3.5  min. This experiment was repeated for each of the three channels. The MBLL was used, as described previously, to convert raw data to $\Delta \mu_{a}$ values for each S-D pair.

**Fig. 4:**
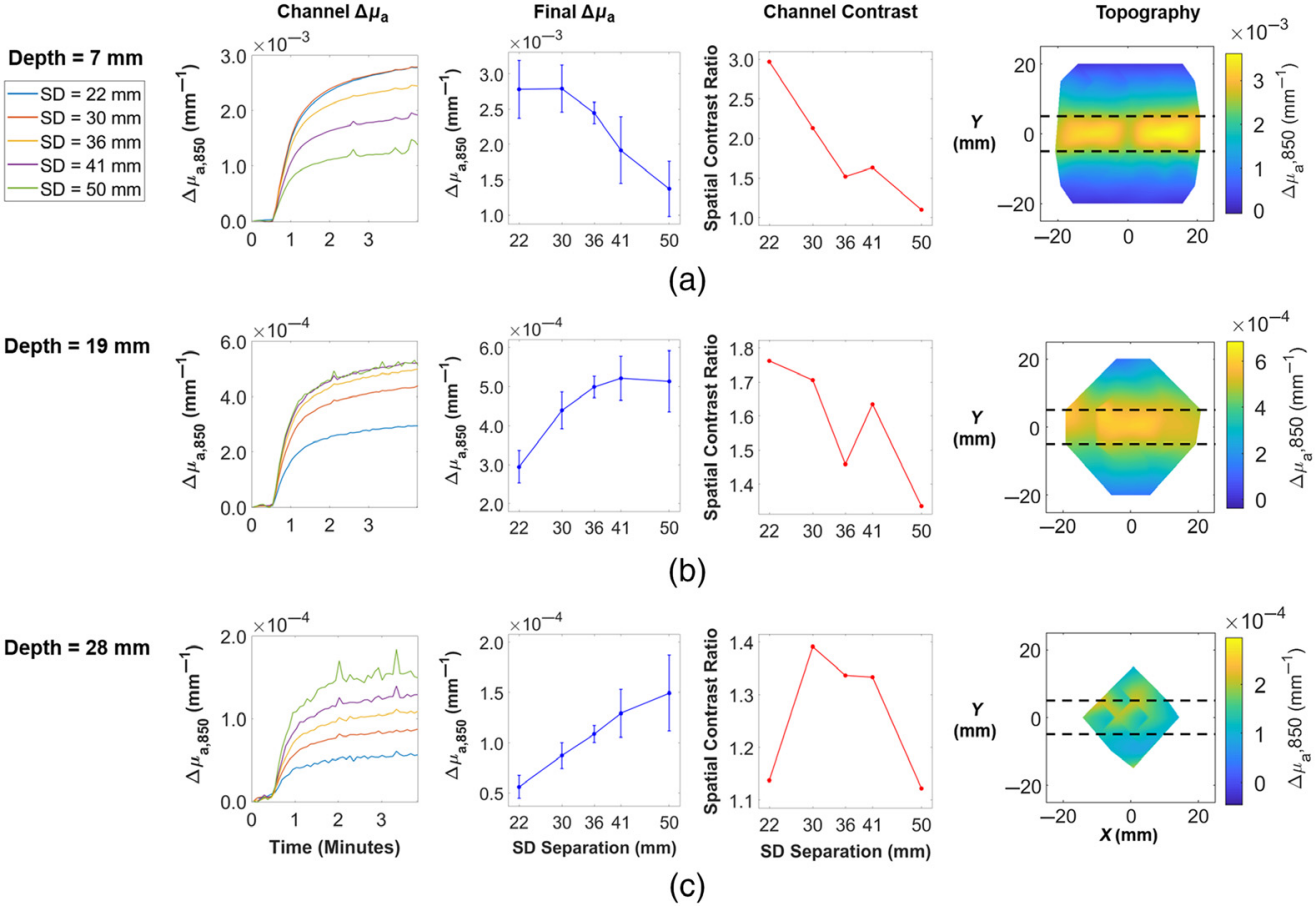
Results from flow phantom experiments for channels whose upper edge were (a) 7 mm, (b) 19 mm, and (c) 28 mm below the background phantom surface. Only 850 nm data are shown. The first column, labeled “Channel $\Delta \mu_{a}$,” depicts the mean $\Delta \mu_{a}$ values as a function of time across all S-D pairs that probe the width of the channel. Flow began after ∼0.5  min. For each S-D separation (going from 22 to 50 mm), the sample sizes (number of S-D pairs within channel) were as follows: n=28, 12, 20, 20, 18. The second column, labeled “Final $\Delta \mu_{a}$,” shows the mean and standard deviation of channel $\Delta \mu_{a}$ values at the final time point of the flow experiment. The third column, labeled “Channel Contrast,” depicts spatial contrast as a function of S-D separation. The final column shows a topographic reconstruction of the final $\Delta \mu_{a}$ values (in x-y space) for each channel depth, each shown for the S-D separation that corresponded to the highest final $\Delta \mu_{a}$. For panel (a), this was 22 mm; for panel (b), this was 41 mm; and for panel (c), this was 50 mm. The colorbar represents $\Delta \mu_{a}$ in units of ${\mathrm{mm}}^{-1}$, and the horizontal dashed lines indicate the true boundaries of the channel (Video 1, mp4, 420 KB [URL: https://doi.org/10.1117/1.JBO.26.6.062708.1]; Video 2, mp4, 500 KB [URL: https://doi.org/10.1117/1.JBO.26.6.062708.2], Video 3, mp4, 457 KB [URL: https://doi.org/10.1117/1.JBO.26.6.062708.3]).

Figure 4 shows results at 850 nm. The first column, labeled “Channel $\Delta \mu_{a}$”, shows time traces of mean $\Delta \mu_{a}$ values across all S-D pairs probing a location within the width of the channel. The location that a given S-D pair is probing is approximated as the midpoint (in x-y space) between the source and detector. There was a rise in $\Delta \mu_{a}$ at all S-D separations at all three channel depths. The second column of Fig. 4, labeled “Final $\Delta \mu_{a}$,” shows the mean and standard deviation of channel $\Delta \mu_{a}$ values at the final time point as a function of S-D separation. From Fig. 4(a), it is apparent that for a depth of 7 mm, the maximum detected change in absorption was captured by the 22-mm separation pairs. Figures 4(b) and 4(c) indicate that for depths of 19 and 28 mm, this maximum change was captured by 41 and 50 mm separation pairs, respectively. So, as expected, absorption contrast in deeper channels was better elucidated by longer S-D separations, due to the deeper penetration of detected photons at these longer separations.

The third column, labeled “channel contrast,” depicts a spatial contrast ratio as a function of S-D separation order. The contrast ratio equals the mean $\Delta \mu_{a}$ across S-D pairs that probe the channel divided by the mean of all pairs whose midpoints lie outside the channel. The spatial contrast for the 7-mm channel depth reached a maximum of 2.97 at an S-D separation of 22 mm, which dropped to 1.10 at a separation of 50 mm. For the 19-mm channel depth, the spatial contrast was overall reduced, with a maximum contrast of 1.76 at 22 mm separation, which reduced to 1.34 at 50 mm separation. For the 28-mm channel depth, the contrast was reduced further, with a maximum of 1.39 at 30 mm separation, which is also comparable to the ratios at 36 and 41 mm. For this channel depth, the minimum spatial contrast was observed at both the 22- and 50-mm separations (1.14 and 1.12). This figure depicts how spatial contrast is a function of both channel depth and S-D separation. Moving down the “channel contrast” column, one can see that as channel depth increased, the maximum achievable spatial contrast decreased. For a given channel depth, spatial contrast varied with S-D separation for a number of reasons. First, depth penetration increases with S-D separation, so spatial contrast was lower for S-D separations whose depth penetration did not reach the channel depth. Second, the approximation of the measurement location as the midpoint between source and detector becomes less accurate as S-D separation increases. For short S-D separations, this midpoint was relatively close to both source and detector, so the entire sample region probed by a given S-D pair was well approximated. At longer S-D separations, however, the midpoints were a less accurate approximation of the region being traversed by photons, and the longer photon path lengths blurred sources of absorption contrast that are smaller than the S-D separation. Relatedly, the coverage of the channel varied for different S-D separations, which is an artifact of the probe design and probe positioning. For example, for some 30- and 41-mm S-D pairs, both the source and detector for a given pair were positioned atop the true channel location. For all 36-mm S-D pairs, however, at least one of the two elements was laterally displaced from the channel, which means a smaller fraction of the photon paths traverse the channel. This explains the dip in spatial contrast ratio seen at 36 mm in Figs. 4(a) and 4(b).

Examples of topographic reconstructions of $\Delta \mu_{a}$ at 850 nm are presented in the final column of Fig. 4. A single reconstruction incorporates only optodes that share the same S-D separation. For each flow phantom experiment, the separation group with the highest mean final $\Delta \mu_{a}$ within the channel (maximum point in second column of Fig. 4) was visualized. In Fig. 4(a), this was 22 mm, in Fig. 4(b), this was 41 mm, and in Fig. 4(c), this was 50 mm. The midpoints between sources and detectors were used to approximate the measurement location of an S-D pair, as mentioned previously, and then a cubic interpolation was performed between these points to generate the colormaps seen in the final column of Fig. 4. Qualitatively, the highest $\Delta \mu_{a}$ values appear to be well localized to the ground truth channel location in Figs. 4(a) and 4(b), whereas spatial localization is less clear in the topographic map in Fig. 4(c). This is consistent with the spatial contrast ratios associated with these reconstructions. Videos of the topographic reconstructions over time for all three flow phantoms can be seen in Videos 1–3, as well as the same figures shown in Fig. 4, but for 750-nm data (Fig. S3 in the Supplemental Material). Tomographic reconstructions were not performed in this study, as it was decided that topographic reconstructions with the MBLL would serve as a simpler, more efficient first analysis of the probe performance, validation, and feasibility testing.

### 3.3 *In Vivo* Validation with Cuff Occlusion

A cuff occlusion experiment was performed on a healthy 25-year-old male volunteer to validate the probe’s ability to quantify hemodynamics *in vivo*. This measurement and all subsequent human subject measurements described were performed on subjects who gave written, informed consent under a protocol approved by the Institutional Review Board at Boston University. For this experiment, a pressure cuff was applied to the upper right arm (extending ∼1 to 6 in. above the elbow), with the wearable probe adhered on the ventral proximal forearm with IV tape. For the first 2.5 min of the experiment, no pressure was applied to the cuff. The cuff pressure was then increased to 200 mmHg, where it remained for an additional 2.5 min. Pressure was then released, and an additional 2.5 min of recovery was monitored at zero pressure. Probe measurements were acquired at 0.33 Hz throughout the duration of the experiment. Beer’s law was used to calculate $\Delta {\mathrm{HbO}}_{2}$ and ΔHHb. The optical properties used to compute the DPF were as follows: $\mu_{a}=0.021{\;\mathrm{mm}}^{-1}$ (750 nm), $\mu_{a}=0.023{\;\mathrm{mm}}^{-1}$ (850 nm) and $\mu_{s}^{\prime }=0.72{\;\mathrm{mm}}^{-1}$ (750 nm), $\mu_{s}^{\prime }=0.67{\;\mathrm{mm}}^{-1}$ (850 nm). These optical properties are typical of the forearm region.^40^^,^^41^

**Fig. 5:**
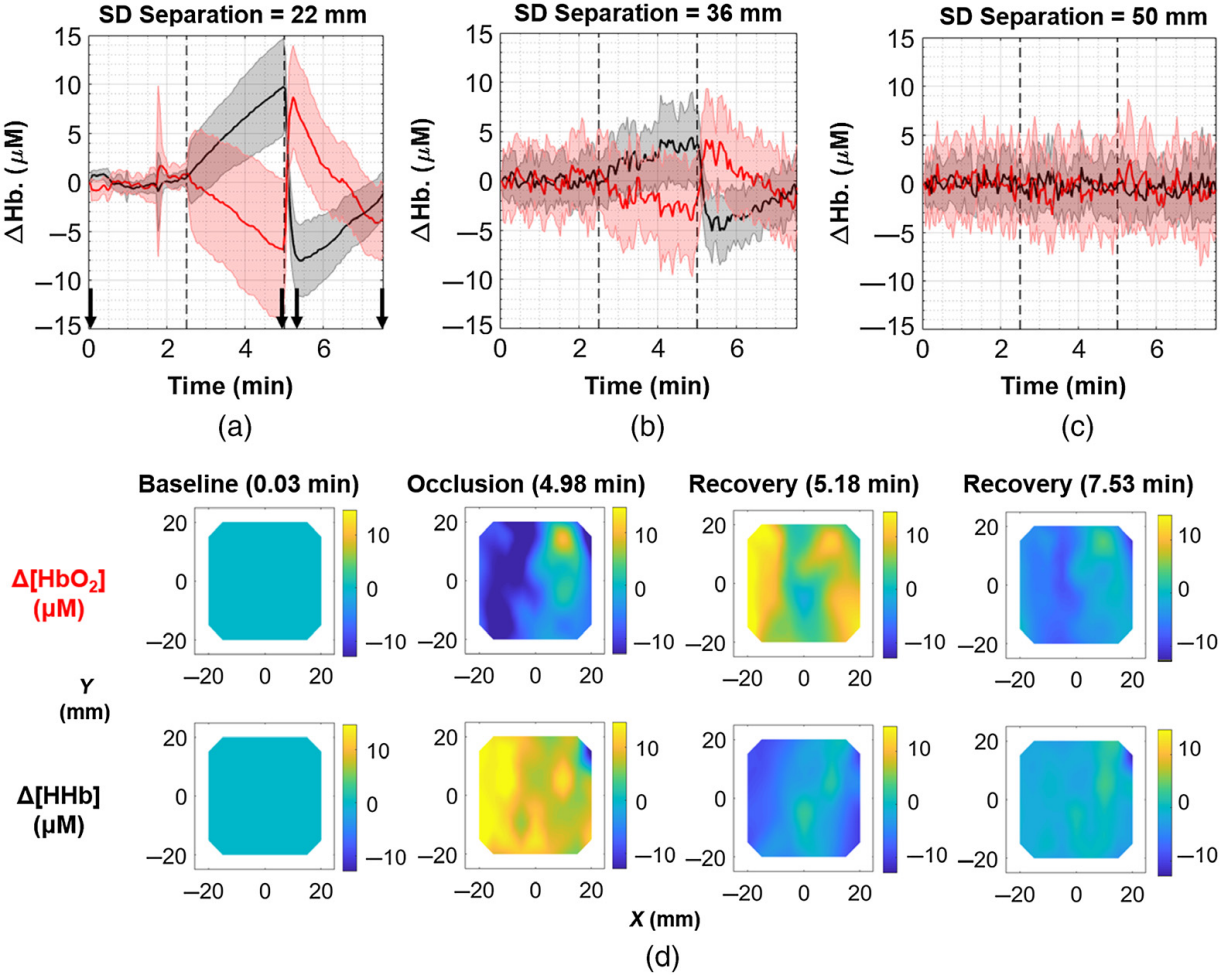
Results from cuff occlusion experiment on healthy volunteer. The top row depicts the mean time traces (solid lines) and standard deviations (shaded regions) for $\Delta {\mathrm{HbO}}_{2}$ (red) and ΔHHb (black) across all channels with a S-D separation of (a) 22 mm (n=72), (b) 36 mm (n=40), and (c) 50 mm (n=24). (d) Topographic reconstructions from 22 mm separations showing changes in $\Delta {\mathrm{HbO}}_{2}$ (upper row) and ΔHHb (lower row) spatially and temporally, with the four pairs of frames referring to the time points designated by the four black arrows in (a). The first column is during baseline, second column just before the end of the occlusion, third column shortly after the end of occlusion, and fourth column the final time point. The colorbar is in units of μM, and each frame represents a 50  mm×50  mm area (Video 4, mp4, 1795 KB [URL: https://doi.org/10.1117/1.JBO.26.6.062708.4]).

Figure 5(a) shows the hemodynamic response to the cuff occlusion at an S-D separation of 22 mm. The red and black solid lines are equal to the mean $\Delta {\mathrm{HbO}}_{2}$ and ΔHHb values across all optodes sharing a 22-mm S-D separation, and the shaded region refers to the standard deviation across these optodes. It is evident that after the cuff pressure is increased, $\Delta {\mathrm{HbO}}_{2}$ decreased while ΔHHb increased, until the pressure was released at the 5-min mark. At this point, $\Delta {\mathrm{HbO}}_{2}$ then rose and ΔHHb fell, over- and undershooting their baseline values, before eventually returning to near-baseline values. Figures 5(b) and 5(c) demonstrate that the magnitude of this response is decreased at longer separations of 36 and 50 mm, respectively. It is unclear if this attenuation with S-D separation is a true physiological phenomenon (i.e., hemodynamic response is greater in superficial tissues), or if the signal is simply too noisy at longer S-D separations due to the presence of more attenuating muscle tissue. One-sample t-tests revealed statistically significant changes in $\Delta {\mathrm{HbO}}_{2}$ and ΔHHb just prior to pressure release at S-D separations of 22, 30, 36, and 41 mm. The means and standard deviations in $\Delta {\mathrm{HbO}}_{2}$ for these S-D separations were −6.91±7.09  μM (p<0.001), −5.93±6.40  μM (p<0.001), −2.24±4.56  μM (p=0.004), and −2.26±4.51  μM (p=0.008). The corresponding mean changes in ΔHHb were 9.76±4.92  μM (p<0.001), 7.99±4.49  μM (p<0.001), 3.62±3.69  μM (p<0.001), and 1.89±3.85  μM (p=0.009).

Figure 5(d) shows topographic reconstructions for 22-mm separation optodes at various time points during the occlusion experiment. While the mean trends from the time traces indicate that $\Delta {\mathrm{HbO}}_{2}$ decreased and ΔHHb increased during the occlusion, the topographic maps at T=4.98  min [the second pair of maps in Fig. 5(d)] show that there is spatial variation of the hemodynamic response across the forearm locations. For example, it is interesting to note that at approximately (x,y)=(10  mm,10  mm), there appears to be a rise in $\Delta {\mathrm{HbO}}_{2}$ in contrast with the surrounding decrease. Video 4 shows these topographic reconstructions at all five S-D separations over the duration of the occlusion.

## 4 Paced Breathing Hemodynamics in Healthy Breast Tissue

### 4.1 Breast Tissue Depth Penetration Modeling

Modeling was performed to quantify the approximate depth penetration of photons at the range of S-D separation orders available with the wearable probe. The photon hitting density (PHD) was calculated for a semi-infinite geometry using the Virtual Photonics Simulator (Virtual Photonics Initiative, Irvine, California) with the analytic solution to the standard diffusion approximation and an isotropic point source. A homogeneous sample was assumed, with the entire sample having identical breast optical properties. The simulated optical properties were as follows: $\mu_{a}=0.0059{\;\mathrm{mm}}^{-1}$ (750 nm), $\mu_{a}=0.0074{\;\mathrm{mm}}^{-1}$ (850 nm) and $\mu_{s}^{\prime }=0.88{\;\mathrm{mm}}^{-1}$ (750 nm), $\mu_{s}^{\prime }=0.78{\;\mathrm{mm}}^{-1}$ (850 nm). These optical properties are typical of breast tissue for premenopausal women,^34^^,^^35^ which corresponds to the age range of the healthy volunteers in the subsequent breast measurements. A g value (anisotropy factor) of 0.8 was assumed, as well as an index of refraction of 1.4. A line profile of PHD perpendicular to the surface of the sample at the midpoint of the source and detector was assessed to quantify depth penetration metrics. Along that profile, the location in depth of peak PHD was determined, as well as locations of 90%, 50%, and 10% of this peak, as described by Peterson et al.^42^

**Fig. 6:**
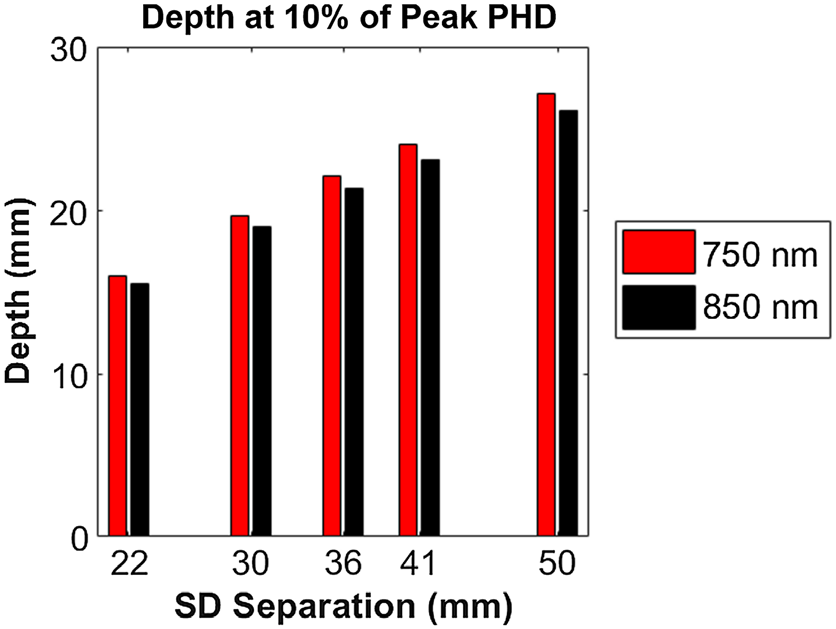
Depth at 10% of peak photon-hitting density, grouped by wavelength and S-D separation.

The results from these simulations are listed in Table S1 in the Supplemental Material. For all simulations, 750- and 850-nm pairs had similar depth penetrations, differing by at most 0.5 mm for S-D separation = 22 mm and by at most 1 mm at S-D separation=50  mm. The peak PHD ranged from 4.3 to 9.5 mm with increasing S-D separation. 50% peak locations ranged from 9.4 to 19 mm across S-D separations, suggesting that sensitivity even at the shortest S-D separations can reach depths around 1 cm and beyond. Figure 6 shows the 10% of peak PHD depths (indicative of the deepest simulated depth penetration), which ranged from 15.5 to 27.1 mm.

### 4.2 Healthy Volunteer Breast Study: Paced Breathing

**Fig. 7:**
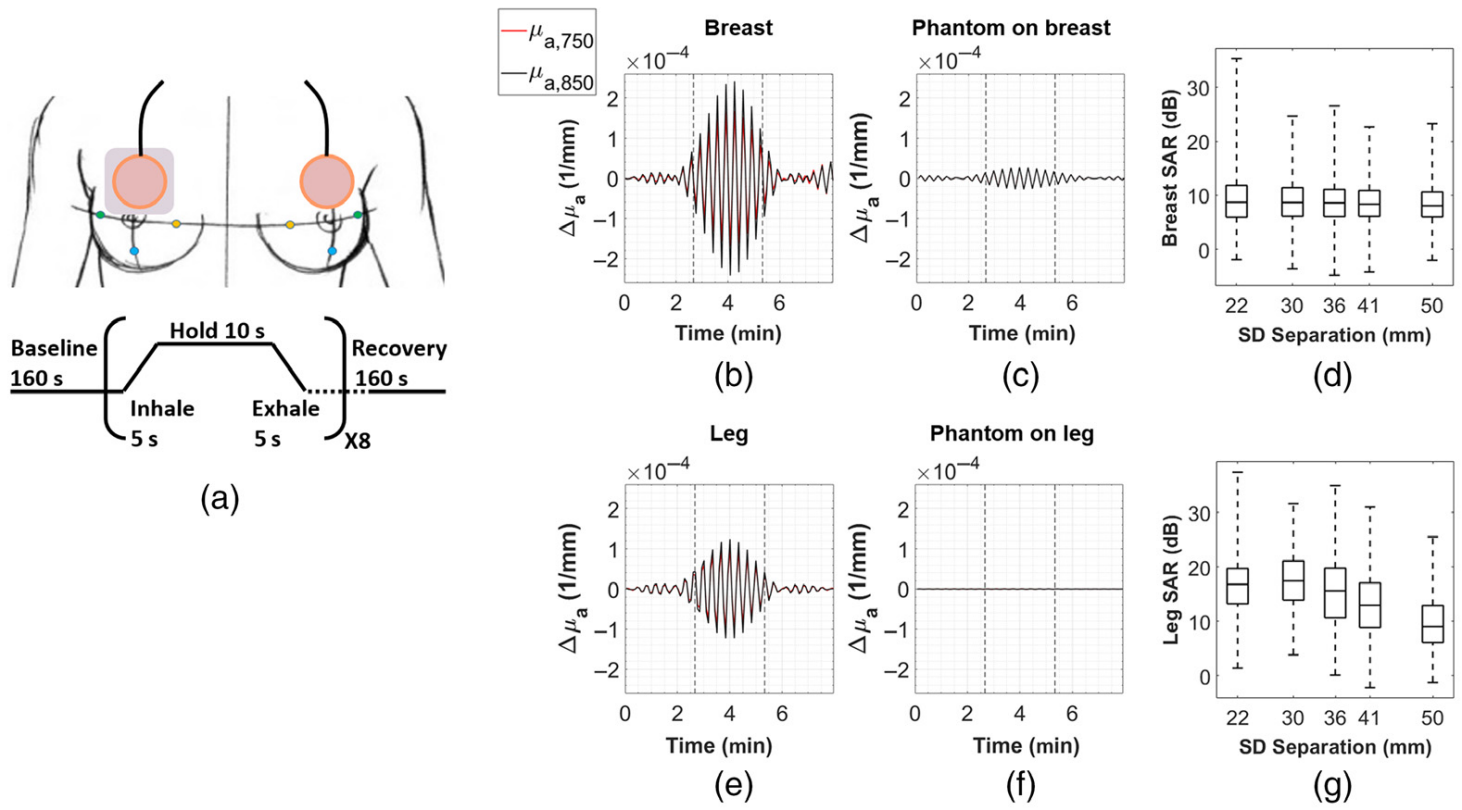
(a) Paced breathing protocol. The gray rectangle between the probe and right breast represents the worn phantom. (b) Representative example of band-pass filtered mean $\Delta \mu_{a}$ time traces from a right breast measurement for 750 nm (red) and 850 nm (black) at 22-mm S-D separation. Each time trace is the mean across n=72 S-D pairs. The vertical dashed lines indicate the start and end of paced breathing at 0.05 Hz. (c) The time traces for the 22-mm S-D separation for the contralateral worn phantom on breast measurement. (d) Boxplots showing the distribution of SAR values across all S-D pairs and trials for the breast measurements, grouped by S-D separation. The horizontal line inside the boxplots indicates the median, and the edges of the box indicate the 25th and 75th percentiles. Whiskers extend to the minimum and maximum values. For breast SARs, total number of S-D pairs from S-D=22  mm to S-D=50  mm that met the QC criteria (and thus are included in this figure) are as follows: n=1616, 620, 804, 616, 418. (e) Representative example of band-pass filtered mean $\Delta \mu_{a}$ time traces from a right leg measurement at 22-mm S-D separation. (f) The time traces for the contralateral worn phantom on leg measurement. (g) Same boxplots as in (d), but for leg SARs. The total number of S-D pairs are: n=1242, 482, 616, 420, 248.

N=4 healthy female subjects were initially measured to determine if paced breathing hemodynamics could be quantified in breast tissue. Subjects were between the ages of 24 and 27. The subjects performed the following protocol: one wearable probe was adhered directly to breast tissue on one side with medical grade IV tape, with the lower edge of the probe centered at the upper edge of the areola. A second probe was adhered to the surface of a relatively small and deformable breast-simulating optical phantom (∼9×9×1.5  cm), and this probe/phantom complex was adhered with additional IV tape to the contralateral breast in the same location. To simulate the interface between probe and breast tissue, this worn optical phantom was fabricated using a recipe that has been previously used to make phantoms with mechanical properties comparable to those of human breast tissue.^43^^–^^45^ Demonstrations of this flexible phantom being adhered to a bust model and being deformed upon contact are shown in Fig. S4 in the Supplemental Material. The tissue-facing side of this worn phantom was lined with a layer of aluminum foil to ensure that photons did not penetrate tissue. The reasoning for this was that there would be no hemodynamic contrast in the phantom, but the probe/phantom complex would still experience torso/chest motion at the breathing frequency and generate optical signal from diffusely scattered light. Signal from the breast and contralateral phantom sides were then compared as a method of quantifying motion artifact. Probe measurements were taken at 0.2 Hz. Measurements were taken for 160 s at baseline (normal breathing), after which volunteers performed eight cycles of paced breathing, with cycles consisting of a 5-s inhale, 10-s hold, and 5-s exhale, yielding a slow breathing rate of 0.05 Hz. After the eight cycles were completed, an additional 160 s of recovery (normal breathing) were monitored. This entire procedure was then repeated two additional times (three trials total). The paced breathing was visually guided by an application on xhalr.org. Figure 7(a) summarizes this procedure. Of the four volunteers, two wore the probe/phantom complex on the right breast, and two wore the probe/phantom complex on the left breast. A breathing rate of 0.05 Hz was selected due to the limited bandwidth (0.1 Hz) of the system.

For each S-D pair from each trial of paced breathing, raw data were first converted to $\Delta \mu_{a}$ using the MBLL as described previously. The initial optical properties used for breast measurements were assumed to be the same as those used for simulations in Sec. 4.1. The optical properties for the worn phantom were measured by the same frequency domain device mentioned previously: $\mu_{a}=0.007{\;\mathrm{mm}}^{-1}$ (750 nm), $\mu_{a}=0.007{\;\mathrm{mm}}^{-1}$ (850 nm) and $\mu_{s}^{\prime }=0.933{\;\mathrm{mm}}^{-1}$ (750 nm), $\mu_{s}^{\prime }=0.765{\;\mathrm{mm}}^{-1}$ (850 nm). An FFT was then performed on the time points that corresponded to the duration of the paced breathing, and the amplitude peak nearest to 0.05 Hz was extracted (always a single bin in the FFT). Three quality control (QC) steps were performed at this stage to remove S-D pairs that did not meet the following criteria: pairs of adjacent 750- and 850-nm S-D pairs had to have (1) peak amplitudes that exceeded a frequency-domain noise floor (approximated as the mean amplitude between 0.06 and 0.09 Hz); (2) peak locations that matched exactly (same frequency bin); and (3) peak amplitudes that exceeded the amplitude at the same frequency bin in the baseline breathing. Each S-D pair from the direct breast measurement that met these criteria was then compared to the same S-D pair on the contralateral worn phantom, and a signal-to-artifact ratio (SAR) was computed according to

$$\mathrm{SAR}=10\cdot \log_{10}(\frac{{\mathrm{Amp}}_{\text{breast},0.05\;\mathrm{Hz}}}{{\mathrm{Amp}}_{\text{phantom},0.05\;\mathrm{Hz}}}).$$ (3)

**Fig. 8:**
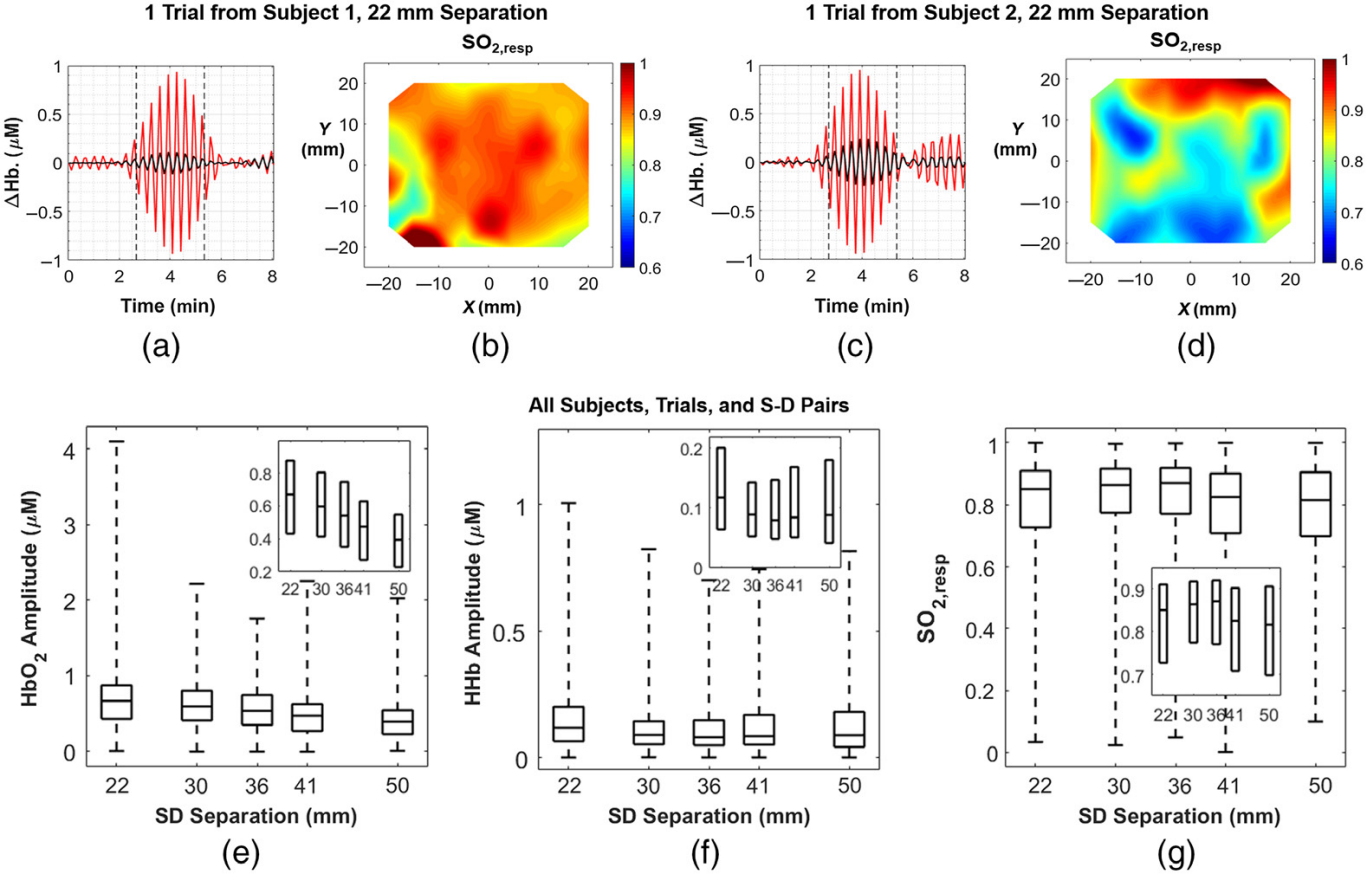
(a) Band-pass filtered mean time traces of ΔHHb (black) and $\Delta {\mathrm{HbO}}_{2}$ (red) for a right breast measurement from a healthy volunteer. Each time trace is the mean value across all 72 channels that have a 22-mm S-D separation. Paced breathing at 0.05 Hz occurred between dashed lines, whereas normal breathing was performed before the first dashed line and after the second. (b) The corresponding ${\mathrm{SO}}_{2,\mathrm{resp}}$ topographic map at 22-mm S-D separation for this trial. (c) The same visualization presented in (a) for a different subject’s left breast. (d) The same visualization presented in (b) for the subject in (c). (e) Distribution of $\Delta {\mathrm{HbO}}_{2}$ amplitudes across all S-D pairs and trials, presented in boxplots. Number of measurement points from S-D=22  mm to S-D=50  mm are as follows: n=795, 309, 388, 303, and 191. (f) Distribution of ΔHHb amplitudes across all S-D pairs and trials. Number of measurement points are as follows: n=696, 273, 344, 260, and 164. (g) Distribution of ${\mathrm{SO}}_{2,\mathrm{resp}}$ values across all S-D pairs and trials. Number of measurement points are as follows: n=683, 272, 330, 255, 146. The horizontal line inside the boxplots indicates the median, and the edges of the box indicate the 25th and 75th percentiles. Whiskers extend to the minimum and maximum values. The insets show zoomed-in version on the boxes without the whiskers to better visualize relationships between hemodynamic metrics and S-D separation.

As a qualitative analysis, TD visualizations were also generated by band-pass filtering the entire time series. A fifth-order Butterworth band-pass filter was used for all time trace filtering with a passband between 0.045 and 0.055 Hz. The filter was generated using the “butter” function in Matlab (version 2018a), and filtering was performed using the “filtfilt” function, which performs forward and backward filtering of the time series to prevent phase distortion introduced by infinite impulse response filters. The smoothing effect of this band-pass filter in both the forward and reverse directions artificially produces the symmetry observed in the time traces in Figs. 7 and 8.

An additional group of N=3 female volunteers (ages 24 to 25) underwent the same protocol, except the probe and a more rigid optical phantom were adhered to the upper quadriceps of contralateral legs. Since leg motion is minimal during breathing compared to chest motion, a strong signal in this location would provide additional evidence that the probe is able to detect physiology related to paced breathing. This more rigid worn phantom had the following measured optical properties: $\mu_{a}=0.005{\;\mathrm{mm}}^{-1}$ (750 nm), $\mu_{a}=0.003{\;\mathrm{mm}}^{-1}$ (850 nm) and $\mu_{s}^{\prime }=0.709{\;\mathrm{mm}}^{-1}$ (750 nm), $\mu_{s}^{\prime }=0.582{\;\mathrm{mm}}^{-1}$ (850 nm). Optical properties of the rectus femoris were used when determining the DPF for leg measurements, which were: $\mu_{a}=0.012{\;\mathrm{mm}}^{-1}$ (750 nm), $\mu_{a}=0.012{\;\mathrm{mm}}^{-1}$ (850 nm) and $\mu_{s}^{\prime }=0.70{\;\mathrm{mm}}^{-1}$ (750 nm), $\mu_{s}^{\prime }=0.65{\;\mathrm{mm}}^{-1}$ (850 nm).^46^

Representative examples of breast and contralateral phantom time traces from one subject are shown in Figs. 7(b) and 7(c), with Fig. 7(b) illustrating a clear rise in amplitude at both wavelengths during the paced breathing phase (between dashed vertical lines). Figure 7(c) shows the relatively low change in amplitude in the worn phantom. These traces are the mean values across all S-D pairs that share a 22-mm S-D separation. Figures 7(e) and 7(f) show the same type of representative examples for a subject from the leg measurement group. Overall, SAR values are relatively high for both breast and leg measurements, and the distributions of this metric in the two groups across all S-D pairs, all trials, and all subjects are shown in Figs. 7(d) and 7(g). For breast measurements, there were 392 S-D pairs per trial × 3 trials per subject × 4 subjects = 4704 total S-D pairs analyzed. The median SARs for each S-D separation, from 22 to 50 mm, were 8.7, 8.7, 8.6, 8.4, and 8.1 dB. It should be noted that 86.6% of all S-D pairs from these breast measurements met the previously described QC criteria [the rest were not included in Fig. 7(d)]. 1.3% of all S-D pairs met the criteria but had a negative SAR, indicating the motion signal was higher than the breast signal. For leg measurements, there were only three subjects, so there were 3528 S-D pairs total. The leg SARs were overall higher than those of breast, with the medians at each S-D separation being 16.8, 17.4, 15.6, 12.9, and 9.0 dB. For the leg, 85.2% of S-D pairs met the QC criteria, whereas 0.2% had a negative SAR.

### 4.3 Healthy Volunteer Breast Study: Hemodynamic Metric Quantification

Data from the same N=4 healthy volunteers were further analyzed to quantify hemodynamic metrics related to paced breathing. Hemodynamics were only quantified for breast data, with worn phantom data excluded (one breast for each of four volunteers). For each S-D pair in each trial of each subject, peak extraction of the $\Delta \mu_{a}$ amplitude at 0.05 Hz was performed in the same manner as described in Sec. 4.2. The same QC steps were employed as well, and, additionally, the noise floor was subtracted from each peak. Pairs of noise-subtracted $\Delta \mu_{a}$ amplitudes at 750 and 850 nm from adjacent optodes were then fit to Beer’s law to extract ΔHHb and $\Delta {\mathrm{HbO}}_{2}$ amplitudes. Oxygen saturation at the respiratory frequency, or ${\mathrm{SO}}_{2,\mathrm{resp}}$, was computed as $\Delta {\mathrm{HbO}}_{2}/(\Delta {\mathrm{HbO}}_{2}+\Delta \mathrm{HHb})$. S-D pairs yielding negative $\Delta {\mathrm{HbO}}_{2}$ or ΔHHb amplitudes, or ${\mathrm{SO}}_{2,\mathrm{resp}}$ values below 0 or above 1 were discarded as an additional QC step.

Figure 8(a) shows overlaid time traces of $\Delta {\mathrm{HbO}}_{2}$ (red) and ΔHHb (black) at an S-D separation of 22 mm for the right breast of a volunteer. It is evident that the amplitude of $\Delta {\mathrm{HbO}}_{2}$, and to a lesser extent ΔHHb, rose during the paced breathing phase of the protocol (between dashed vertical lines) compared to the baseline and recovery phases. Figure 8(b) shows a topographic reconstruction of ${\mathrm{SO}}_{2,\mathrm{resp}}$ for this same subject at 22 mm separation, generated using data from the entire duration of the 0.05-Hz paced breathing, showing high saturation throughout most of sampled region. In contrast, Figs. 8(c) and 8(d) show the same time traces and topographic map for another subject whose ${\mathrm{SO}}_{2,\mathrm{resp}}$ was overall lower and more heterogeneous. Interestingly, the time trace for this subject appears to show a rise in amplitude toward the end of the recovery phase as well, which may be an artifact or a residual tendency to continue the paced breathing. Distributions of $\Delta {\mathrm{HbO}}_{2}$ amplitude, ΔHHb amplitude, and ${\mathrm{SO}}_{2,\mathrm{resp}}$ are shown in Figs. 8(e)–8(g), respectively, across all S-D pairs and trials from the N=4 subjects. For these distributions, there were 2352 ΔHHb and 2352 $\Delta {\mathrm{HbO}}_{2}$ values analyzed. $\Delta {\mathrm{HbO}}_{2}$ amplitude appears to decrease with S-D separation. The median $\Delta {\mathrm{HbO}}_{2}$ amplitudes for each S-D separation, from 22 to 50 mm, were 0.67, 0.60, 0.54, 0.47, and 0.39  μM. 84.4% of all $\Delta {\mathrm{HbO}}_{2}$ measurements met the QC criteria described previously, with excluded measurements being removed from these distributions. The median ΔHHb amplitudes were 0.12, 0.09, 0.08, 0.08, and 0.09  μM. For ΔHHb, 73.9% of all measurements passed the QC criteria. While error in these measurements was not quantified directly, precision in $\Delta \mu_{a}$ for each wavelength and each S-D separation (derived from the previous drift measurement in Sec. 3.1), the extinction coefficients, and rules of error propagation were used to estimate uncertainty. For $\Delta {\mathrm{HbO}}_{2}$, the uncertainties at each S-D separation were 0.03, 0.02, 0.03, 0.03, and 0.06  μM. For ΔHHb, they were 0.02, 0.02, 0.01, 0.02, and 0.03  μM.

The median ${\mathrm{SO}}_{2,\mathrm{resp}}$ values were 0.85, 0.86, 0.87, 0.83, and 0.82. For ${\mathrm{SO}}_{2,\mathrm{resp}}$, 71.7% of all measurements passed QC criteria. The computation of oxygen saturation metrics at AC frequencies generally assumes that $\Delta {\mathrm{HbO}}_{2}$ and ΔHHb are in-phase as a result of blood volume oscillations. While many measurements were in phase, the mean phase difference across all measurements that met the QC criteria was 19±50  deg.

## 5 Discussion

We have described here a wearable CW diffuse optical probe with 512 unique S-D pairs ranging from 10 to 54 mm in S-D separation. Furthermore, we demonstrated that hemodynamics related to the respiratory cycle can be quantified noninvasively *in vivo* in human subjects. Probe performance was characterized through measurements on tissue-simulating phantoms and validated *in vitro* through measurements on spatially complex flow phantoms and *in vivo* through a cuff occlusion measurement on a healthy volunteer.

There have been a number of portable and wearable NIRS devices developed for various medical applications. As far back as 20 years ago, Chance et al. developed a portable NIRS device with 16 channels that was tethered via cable to external control hardware and continued to push the technology forward with the fabrication of a single channel, battery-powered flexible device for hematoma detection, which afforded increased portability and improved tissue contact.^47^^,^^48^ A wireless, wearable NIRS device has also been fabricated for the purpose of measuring cortical activation.^49^ A number of commercial wireless, wearable devices are also now available, such as the Portamon from Artinis (Einsteinweg, The Netherlands) and the MOXY from Fortiori Design (Hutchinson, Minnesota).^17^^,^^18^ These devices have few optodes and are primarily used to quantify point measurements of oxygenation. A wired ambulatory DOT device with 64 S-D pairs has shown to be able to track hemodynamics in muscles,^19^ and another 128-channel wired NIRS device has been developed for monitoring brain activity, although the configuration of this device is optimized to be fixed around human head.^20^ Compared to prior wearable NIRS devices, our probe has the greatest number of S-D pairs at 512, offering both high area coverage and high optode-density. While lower optode-density devices typically offer better tissue contact and flexibility, our rigid-flex design enables this high spatial sampling while preserving this flexibility. Also, while this device is designed for use on breast tissue, its flexibility is such that it could be extended to almost any other anatomical location that does not require extreme flexion. However, acquisition speed for other devices is generally higher, with the wired ambulatory DOT system showcasing speeds up to 250 Hz, for example.

The experiments in Secs. 3.2 and 3.3 validate the utility of this device. The flow phantom experiments demonstrate how the range of S-D separations and optode positions offered allow for sensitivity to absorption contrast at a range of axial and lateral positions. Furthermore, the cuff occlusion experiment confirms that the probe can track the expected hemodynamic response to a perturbation *in vivo*.

The healthy volunteer study presented in Secs. 4.2 and 4.3 demonstrates that paced breathing hemodynamics are likely quantifiable in breast tissue. The comparison between breast measurements and contralateral worn deformable phantom measurements suggests that the observed oscillations during paced breathing reflect actual physiology, although, as shown in Fig. 7(c), motion artifact at the breathing rate likely contributes to this signal as well. While the deformable phantom attempts to mimic the mechanical properties of breast tissue, probe contact with tissue and phantom may still differ, especially considering the variation in breast composition, shape, and size, and the motion artifact may be underestimated. Oscillations observed in leg measurements at the breathing rate, whose motion should not correlate with breathing-induced motion, confirm that the probe is sensitive to such hemodynamics *in vivo*. As expected, leg SAR values generally exceeded breast SAR values, which is likely in part due to the decreased motion artifact in the leg region. That said, distributions of breast SAR values overlapped with leg SAR distributions, which further supports the notion that these hemodynamics are measurable in breast tissue.

The ability to quantify the amplitudes of $\Delta {\mathrm{HbO}}_{2}$ and ΔHHb, and relatedly to derive ${\mathrm{SO}}_{2,\mathrm{resp}}$, offers a new slate of optical biomarkers in breast tissue that may be able to identify tumor contrast in breast cancer patients. The effects of respiratory-induced changes in peripheral blood vessels are detailed by Franceschini et al. and Wolf et al.^25^^,^^26^ Briefly, during inspiration, a decrease in intrathoracic pressure creates a pressure gradient between extrathoracic vessels and the intrathoracic vessels and the heart. As veins are ∼20 times more compliant than arteries, this pressure difference primarily results in volume changes in venous compartments, decreasing venous blood volume while increasing venous return to the heart. Franceschini et al. note that due to the presence of vein valves, the decrease in venous return during expiration does not match the increase during inspiration, creating a respiratory pump effect that modulates central venous pressure and, resultantly, venous blood volume, at the respiratory rate.

The ${\mathrm{SO}}_{2,\mathrm{resp}}$ parameter may be of particular interest in breast cancer treatment monitoring, for it may correlate with or be equal to the venous oxygen saturation ${\mathrm{SvO}}_{2}$. While tissue oxygen saturation, or ${\mathrm{StO}}_{2}$, has been monitored in breast tissue during NAC,^7^^,^^11^ ${\mathrm{SvO}}_{2}$ has yet to be quantified in breast or in breast tumors. The ${\mathrm{SO}}_{2,\mathrm{resp}}$ values calculated in breast tissue for the N=4 healthy subjects in this volunteer study are generally higher than reported values from previous noninvasive NIRS measurements of ${\mathrm{SvO}}_{2}$, with median breast values ranging from 82% to 87%. Franceschini et al.^25^ reported baseline ${\mathrm{SvO}}_{2}$ values of 75% to 78% when probing the vastus medialis muscle (inner thigh) of a healthy adult volunteer (age not stated). Wolf et al.^26^ reported mean cerebral ${\mathrm{SvO}}_{2}$ values of 73±9% across 15 neonates, and Kainerstorfer et al.^23^ reported mean cerebral ${\mathrm{SvO}}_{2}$ values of 66±14% across three healthy adults (ages 25 to 49). Closer to our extracted values, Lynch et al.^24^ reported mean ${\mathrm{SvO}}_{2}$ values of 79±7% on forehead measurements from five healthy volunteers (age not stated). However, there are some important differences to note between previous *in vivo* NIRS measurements of ${\mathrm{SvO}}_{2}$ and the breast measurements presented in this work. First, the sample sizes are all small, and the age groups are different across studies, with some age groups unknown. Another important distinction between our study and the previous ones described is the breathing rate. The breathing rate in the neonate study was 0.5 Hz. For the vastus medialis measurement by Franceschini et al.^25^ and forehead measurements by Lynch et al.,^24^ the breathing rates were 0.25 and 0.18 Hz, respectively, which are close to spontaneous breathing rates. The study by Kainerstorfer et al.^23^ had subjects fix their breathing rate at 0.1 Hz. The breathing rate in our study of 0.05 Hz is the lowest rate of any of these studies. It may be possible that this slow breathing frequency is producing different physiological effects than spontaneous or near-spontaneous breathing. More exploration of the paced breathing frequency is needed to clarify this difference. Lastly, the extinction spectra source is not always reported, and the usage of different spectra across studies could lead to variation in ${\mathrm{SvO}}_{2}$.

It should also be reiterated that a possible limitation, or complication, to our ${\mathrm{SO}}_{2,\mathrm{resp}}$ measurements relates to the relative phase of the $\Delta {\mathrm{HbO}}_{2}$ and ΔHHb. As stated before, blood volume oscillations should produce in-phase hemodynamic changes. The spread of relative phases observed in this study may suggest there is some contribution from blood flow changes, which are known to produce out-of-phase oscillations.^23^ It is also possible that phase extraction was inaccurate in some cases due to relatively low ΔHHb oscillations. Further exploration of relative phase will be a focal point of future studies to better understand the physiology related to these respiratory maneuvers.

While we are not aware of other paced breathing studies aimed at human breast hemodynamics, there have recently been several studies exploring fast tumor hemodynamics in response to breath holds, occurring on the order of seconds to tens of seconds.^14^^–^^16^ One study found that breath hold hemodynamic responses were able to predict pCR at the 2-week point of NAC with strong negative predictive value (94%) and moderate positive predictive value (71%).^16^ A preclinical study of a rat breast cancer model yielded results consistent with these findings, demonstrating that changes in ${\mathrm{HbO}}_{2}$ during simulated breath holds could differentiate treatment and control groups and that optical changes preceded tumor volume changes.^50^ Another clinical study demonstrated that compression-induced hemodynamics were able to differentiate responders from nonresponders within the first month of chemotherapy.^51^

This leads to an important limitation of this system, that being acquisition speed. With two probes being sequentially scanned at 0.2 Hz, bandwidth is greatly limited to just 0.1 Hz, preventing measurements at a wider range of frequencies, including those closer to spontaneous breathing. While subjects were able to perform the breathing protocol without difficulties, 0.05 Hz may be difficult for other subjects, for only a small sample size was measured here. The quantification of paced breathing hemodynamic metrics in breast tissue at 0.05 Hz still is significant, but more information about the underlying physiology, and perhaps greater prognostic potential, could be achieved with measurements at different breathing frequencies. In addition, being a CW device, analysis is limited to relative quantification of optical properties and chromophore concentrations. Therefore, assumptions about initial optical properties of breast (and other various anatomical regions measured, such as leg and forearm) had to be made based on literature values. With respect to subject population of this study, the number and age range of subjects were both small, limiting conclusions that can be made about a broad population of healthy women. It is important to note that breast location and breast size may impact the quantification of these hemodynamic parameters. While these factors were not explored in this initial study, we plan to assess them going forward in a larger subject population. Also, oscillations in ΔHHb may be below the noise floor of the probe in some instances. This could be a product of poor tissue contact for some optodes, which may increase noise and motion artifact. Alternative adhesives as well as more flexible silicone probe housings will be explored as methods of optimizing tissue contact.

We have demonstrated the utility of a custom high optode-density wearable diffuse optical probe through performance characterization, validation, and breast measurements. Optodes with S-D separations ranging from 22 to 50 mm had high SNR, high precision, and low drift, and validation experiments with flow phantoms and a cuff occlusion confirmed the ability to quantify absorption contrast, both spatially or temporally. Breast measurements with a worn phantom suggested that while motion artifact contributed to the measured signal (and may be underestimated), optical signal is reflective of actual hemodynamics, demonstrating the feasibility of quantifying breathing hemodynamics such as $\Delta {\mathrm{HbO}}_{2}$ and ΔHHb amplitudes as well as ${\mathrm{SO}}_{2,\mathrm{resp}}$ in breast tissue.
