# Supplementary material for: High optode-density wearable diffuse optical probe for monitoring paced breathing hemodynamics in breast tissue
Source: J Biomed Opt. 2021 Jun 2;26(6):062708. doi: 10.1117/1.JBO.26.6.062708 (PMC8170390; doi:10.1117/1.JBO.26.6.062708)
Supplement: Supplementary file 1 [file JBO_026_062708_SD001.pdf]

Supplementary Information

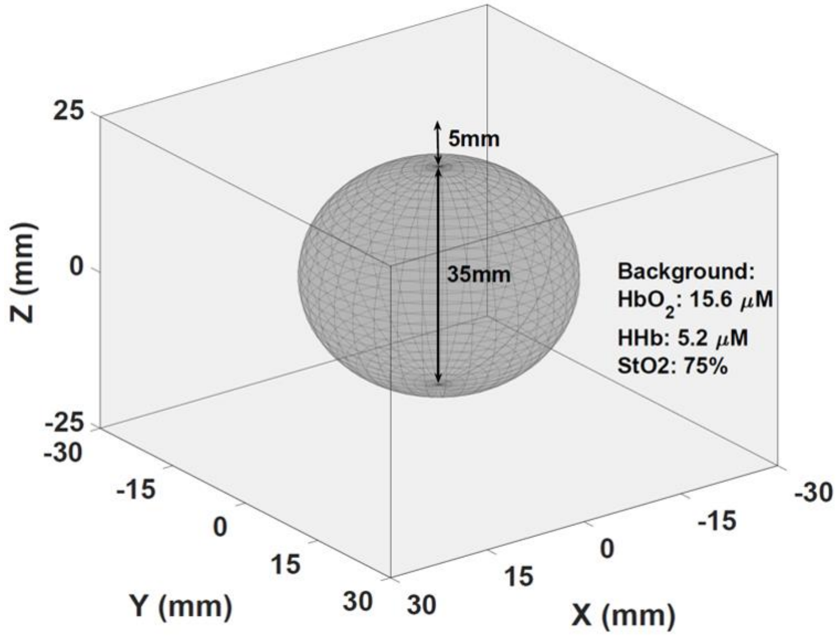

Supplementary Fig. S1 Simulation geometry utilized for optode configuration comparisons. The inclusion mimics a 35 mm diameter breast tumor embedded in healthy breast tissue.

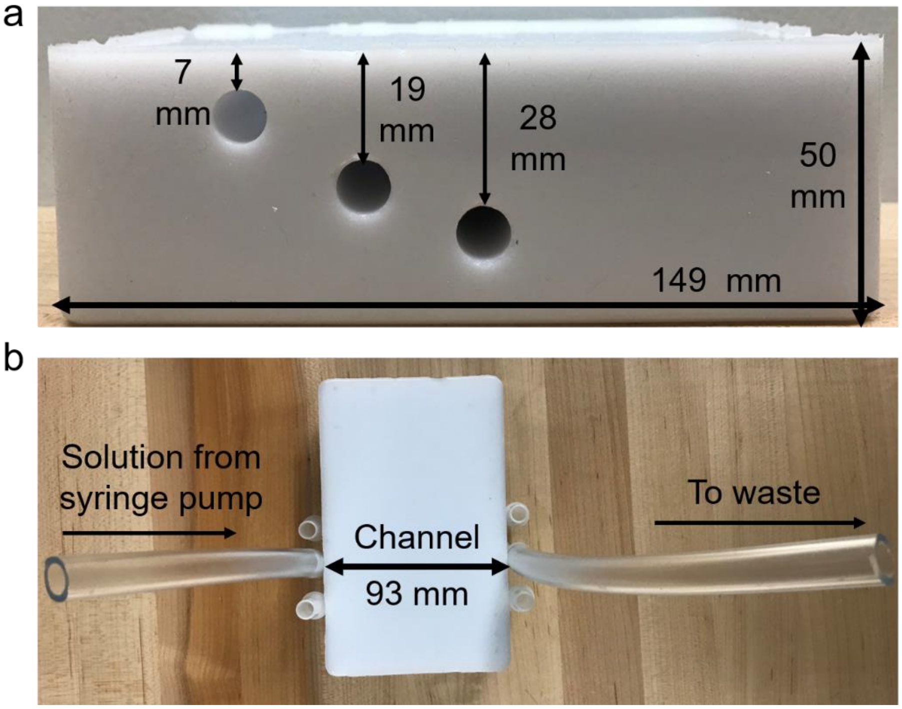

Supplementary Fig. S2 a) Side view of the silicone channel phantom with three channels at varying depths. The nominal depth refers to the distance between the surface of the phantom and the upper edge of the channel. b) Top view of the silicone phantom with right-angle tubing adapters connected to each channel. These enable the channels to be pre-filled with the first solution. Tubing is connected to the adapters for one of the three channels, showing how the syringe pump can dispense the second solution into the channel. Not shown here are the syringe pump, syringe, and waste container.

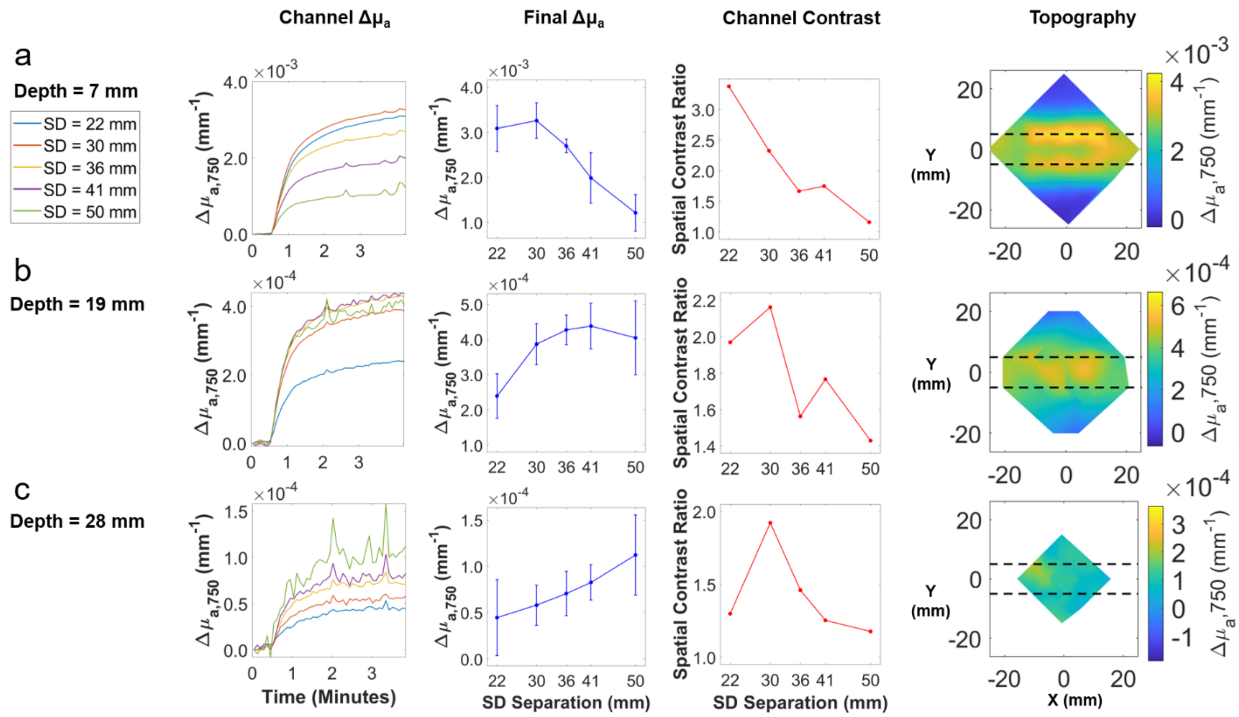

Supplementary Fig. S3 Results from flow phantom experiments for channels whose upper edge were (a) 7 mm, (b) 19 mm, and (c) 28 mm below the background phantom surface. Only 750 nm data is shown. The first column, labeled "Channel $\Delta\mu_a$", depicts the mean $\Delta\mu_a$ values as a function of time across all S-D pairs that probe the width of the channel. Flow began after ~0.5 minutes. For each S-D separation (going from 22 mm to 50 mm), the sample sizes (number of S-D pairs within channel) were as follows: n=28, 12, 20, 20, 18. The second column, labeled "Final $\Delta\mu_a$", shows the mean and standard deviation of $\Delta\mu_a$ values at the final time point of the flow experiment. The third column, labeled "Channel Contrast", depicts spatial contrast as a function of S-D separation. The final column shows a topographic reconstruction of the final $\Delta\mu_a$ values (in x-y space) for each channel depth, each shown for the S-D separation that corresponded to the highest final $\Delta\mu_a$. For (a), this was 22 mm, for (b) this was 41 mm, and for (c), this was 50 mm. The colorbar represents $\Delta\mu_a$ in units of mm^{-1}, and the horizontal dashed lines indicate the true boundaries of the channel.

Supplementary Table S1 Depth penetration estimates using photon hitting density (PHD) from simulations

**S-D Separation**	**22 mm**		**30 mm**		**36 mm**		**41 mm**		**50 mm**	
**Wavelength**	**750 nm**	**850 nm**	**750 nm**	**850 nm**	**750 nm**	**850 nm**	**750 nm**	**850 nm**	**750 nm**	**850 nm**
**Depth at PHD Peak (mm)**	**4.5**	**4.3**	**6.1**	**5.8**	**7.2**	**6.9**	**8.1**	**7.7**	**9.5**	**9.1**
**Depth at 90% Peak (mm)**	**6.3**	**6**	**8.3**	**8**	**9.7**	**9.3**	**10.8**	**10.3**	**12.5**	**12**
**Depth at 50% Peak (mm)**	**9.8**	**9.4**	**12.5**	**12**	**14.3**	**13.8**	**15.7**	**15.1**	**19**	**17.4**
**Depth at 10% Peak (mm)**	**16**	**15.5**	**19.7**	**19**	**22.1**	**21.3**	**24**	**23.1**	**27.1**	**26.1**

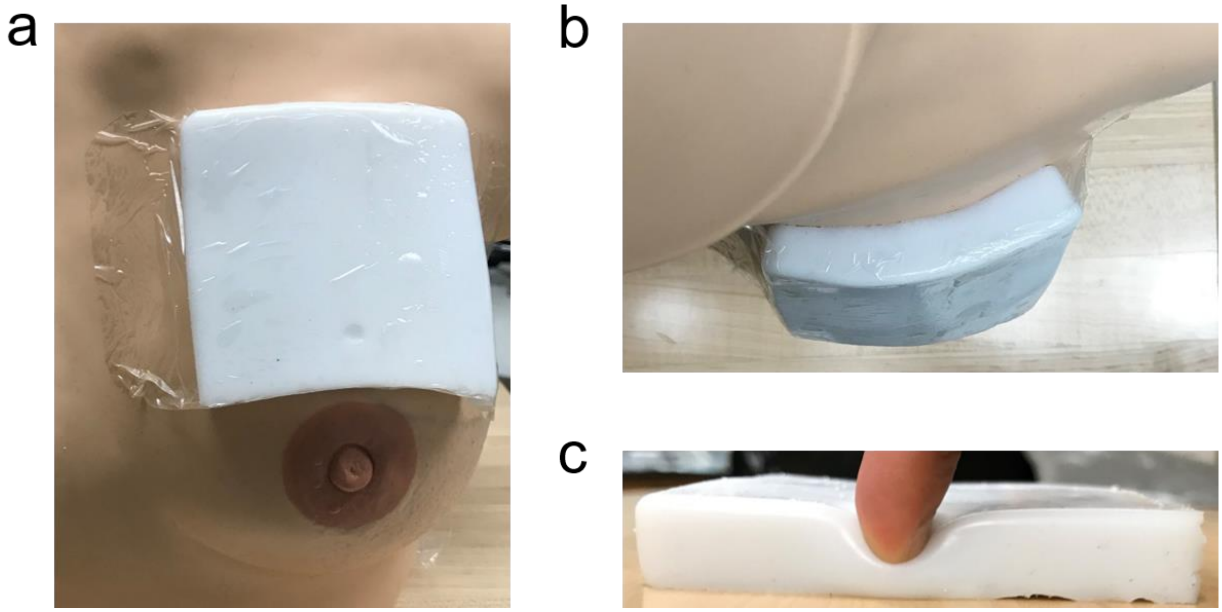

Supplementary Fig. S4 Depictions of the deformable phantom described in Section 4.2. (a) View of phantom adhered to bust model. b) Top-view bust model/phantom shown in (a). c) Demonstration of the deformability of the phantom, with a finger making an indent into the phantom by pressing.

Supplementary Video S1:

This video displays 10 panels in a 2 x 5 format. The upper row shows topographic reconstructions of $\Delta\mu_a$ over time for the 750 nm S-D pairs at the five S-D separations for the 7 mm depth flow phantom experiment. From left to the right, the S-D separations are 22 mm, 30 mm, 36 mm, 41 mm, and 50 mm. The bottom row shows the topographic reconstructions of $\Delta\mu_a$ for the 850 nm S-D pairs. The colorbar in each row applies to all S-D separations for that row.

Supplementary Video S2:

The format of this video is the same as in Supplementary Video 1, except this video displays topographic reconstructions for the 19 mm depth flow phantom experiment.

Supplementary Video S3:

The format of this video is the same as in Supplementary Video 1, except this video displays topographic reconstructions for the 28 mm depth flow phantom experiment.

Supplementary Video S4:

This video displays 10 panels in a 2 x 5 format. The upper row shows topographic reconstructions of ΔHbO_2 over time at the five S-D separations during the cuff occlusion experiment. From left to the right, the S-D separations are 22 mm, 30 mm, 36 mm, 41 mm, and 50 mm. The bottom row shows the topographic reconstructions of ΔHHb. The colorbar in each row applies to all S-D

separations for that row. The time is shown above all panels in addition to the phase of the experiment (baseline, perturbation, recovery).
